# A Retrospective Assessment of Thrombophilia in Pregnant Women with First and Second Trimester Pregnancy Loss

**DOI:** 10.3390/ijerph192416500

**Published:** 2022-12-08

**Authors:** Olivera Iordache, Diana Maria Anastasiu-Popov, Doru Mihai Anastasiu, Marius Craina, George Dahma, Geanina Sacarin, Carmen Silaghi, Cosmin Citu, Razvan Daniluc, Denisa Hinoveanu, Bogdan Feciche, Felix Bratosin, Radu Neamtu

**Affiliations:** 1 Department of Obstetrics and Gynecology, “Victor Babes” University of Medicine and Pharmacy Timisoara, 300041 Timisoara, Romania; 2 Department of Urology, Satu-Mare County Emergency Hospital, Strada Ravensburg 2, 440192 Satu-Mare, Romania; 3 Methodological and Infectious Diseases Research Center, Department of Infectious Diseases, “Victor Babes” University of Medicine and Pharmacy, 300041 Timisoara, Romania

**Keywords:** miscarriage, pregnancy loss, coagulation factors, venous thromboembolism

## Abstract

Recurrent Pregnancy Loss (RPL) affects between 1% to 5% of women of reproductive age. It is widely believed that RPL is a complex disorder that is influenced by chromosomal abnormalities, genetic mutations, uterine anatomic deformity, endocrine dysfunction, immunologic factors, infections, and the environment. Thrombotic disorders are a frequent cause of RPL, accounting for almost half of all cases; however, in the rest of the cases, the cause of RPL remains unclear. Therefore, in this study, it was planned to determine the genetic mutations involved in RPL during the first and second trimester of pregnancy. An observational retrospective cohort study was conducted in 2021, collecting data from 157 first trimester miscarriages and 54 s trimester pregnancies. All patients with a panel of laboratory and genetic analysis for thrombophilia were included for data analysis. It was observed that four factors were significantly more prevalent in one of the groups. Factor V Leiden (FVL) homozygosity and antiphospholipid syndrome (APS) antibodies were statistically significantly more common in pregnant women who suffered first trimester pregnancy losses. On the other hand, Protein C deficiency and Glycoprotein Ia polymorphism were statistically significantly more frequent in the second trimester group. The strongest independent risk factors for first trimester pregnancy loss were FVL and prothrombin (PT) compound mutations (OR = 3.11), followed by FVL homozygous mutation (OR = 3.66), and APS antibodies (OR = 4.47). Regarding second trimester pregnancy loss risk factors, the strongest were FVL and PT compound (OR = 3.24), followed by Glycoprotein Ia polymorphism (OR = 3.61), and respectively, APS antibodies (OR = 3.85). Numerous thrombophilic risk factors for early and late pregnancy loss have been found, including several mutations that seem to occur more often either during the first or the second trimester. Even though we are aware of risk-free and efficient diagnostics for thrombophilia abnormalities, no intervention has been proved to be clearly successful after the detection of these variables.

## 1. Introduction

Recurrent pregnancy loss (RPL) is a frequent reproductive condition that occurs in between 1% and 3% of patients. Existing guidelines describe RPL as two or more miscarriages documented by ultrasonography or histopathology, or three or more consecutive pregnancy losses before 20 weeks of pregnancy [1,2]. Although about 2% of pregnant women suffer two successive miscarriages, only up to 1% encounter three consecutive miscarriages. RPL is multifactorial and its cause is poorly understood. Many variables, such as maternal age, chromosomal abnormalities, uterine morphological abnormalities, endocrine illnesses, thrombophilia, infections, and autoimmune disorders, contribute to the etiology of RPL, although they are often undiscovered [3,4].

Hemostasis changes during pregnancy are physiological, preparing the pregnant woman during the peripartum period in case of blood loss, as the physiologic hemostasis transcends into a hypercoagulable state [5]. Yet, they may also predispose both the mother and the neonate to difficulties during pregnancy, continuing for at least 12 weeks after pregnancy [6]. Pre-eclampsia, placental abruption, fetal growth restriction (FGR), miscarriage or recurrent pregnancy loss (RPL), mortality from intrauterine causes, and stillbirth are all potential dangers to the maternal-fetal dyad [7]. Although the risks of RPL are fundamentally greater in women who have acquired or inherited thrombophilia, systematic screening for these diseases is not usually suggested in the absence of venous thromboembolism [8]. It is difficult to determine whether screening women who have experienced complications during pregnancy is beneficial because of the gaps in our understanding of the factors that contribute to these complications during pregnancy and the absence of evidence supporting interventions that are effective [9].

The presence of placental vascular thrombosis has led researchers to hypothesize that thrombophilia may be the cause of placental insufficiency that creates an unfavorable environment for the fetus to develop, often causing miscarriage or pregnancy complications in later stages of pregnancy [10,11,12,13]. Late pregnancy complications include pre-eclampsia, fetal growth restriction, preterm delivery, and stillbirth [14]. These issues are directly related to circulatory complications in the placenta that are induced by antiphospholipid syndrome (aPL) and culminate in placental insufficiency [15].

The aPL is another type of thrombophilia that is diagnosed in many pregnant patients that are affected by miscarriage [16]. Often, this condition is diagnosed when the patient suffers an episode of thrombosis or in 10–20% of women with a history of miscarriage [17]. The diagnostic criteria for this condition are stringent, and patients must be identified with anticardiolipin antibodies (aCL), lupus anticoagulant (LA), or anti—2-glycoprotein I antibodies (a2-GPI), which must have persisted for two or more separate occasions, at least 12 weeks apart from each other [18]. Guidelines suggest testing for antiphospholipid antibodies when a woman has had two or more miscarriages [19]. It is advised that patients with RPL have thyroid function testing as well as an examination of their uterine anatomy; however, it is not specified how many pregnancy losses should prompt this suggestion. Additionally, it is not generally suggested to perform parental karyotyping, only after conducting an individual risk assessment should the possibility be considered, given the extremely low likelihood of discovering an abnormality [20]. Screening for hereditary thrombophilia is not often suggested for couples when one partner has RPL. This is because there is only a weak correlation between RPL and hereditary thrombophilia, and there is currently no therapy that is supported by evidence.

Nevertheless, there is still not enough evidence in the existing research to demonstrate that there was a substantial connection between a2-GPI and pregnancy loss, including many other mutations [21]. Considering the continuous concerns and unanswered questions regarding recurrent pregnancy loss, the current study aimed to compare pregnant women affected by first trimester pregnancy loss with pregnant women who suffered from recurrent second trimester pregnancy loss. A secondary aim was to determine the factors that contributed the most towards miscarriage.

## 2. Materials and Methods

### 2.1. Study Design and Settings

An observational retrospective cohort study was conducted between January 2020 and January 2022 with patients who enrolled in the study during that time period at the University Clinic of Obstetrics and Gynecology “Bega” affiliated with the “Victor Babes” University of Medicine and Pharmacy from Timisoara. The research population as well as the pertinent characteristics were obtained from the clinic’s outpatient population-based administrative database. Patient data available in the digital and paper records included chief complaints, demographic information, laboratory analysis data, and existing interventions, which were protected by privacy regulations and patients’ consent. These records were reviewed by licensed medical professionals who were taking part in the current study.

Bega Clinic, as an auxiliary of Timis County Emergency Clinical Hospital “Pius Brinzeu”, works under the laws of the Local Commission of Ethics that approves Scientific Research and operates in accordance with: (1) the Article 167 of Law No. 95/2006, Art. 28, Chapter VIII of Order 904/2006; (2) the EU GCP Directives 2005/28/EC; (3) the International Conference on Harmonisation of Technical Requirements for Registration of Pharmaceuticals for Human Use (ICH); and (4) with the Declaration of Helsinki for recommendations guiding medical practice. The current investigation was given approval on January 20, 2022, identified with the number 27.

### 2.2. Participants and Definitions

Women with a history of pregnancy loss were included in the current study based on the definition by World Health Organization of pregnancy loss, also known as miscarriage [22]. Miscarriage or spontaneous abortion is the most prevalent type of pregnancy loss, being described as the loss of a pregnancy before 20 weeks of gestation by the American College of Obstetricians and Gynecologists (ACOG) [23]. Recurrent pregnancy loss is defined according to the American Society for Reproductive Medicine (ASRM) and European Society of Human Reproduction and Embryology (ESHRE) as two or more miscarriages, while the Royal College of Obstetricians and Gynaecologists (RCOG) defines it as three or more consecutive miscarriages. After 20 weeks of gestation, the loss of pregnancy is known as fetal demise [24]. It is estimated that a quarter of all pregnancies and ten percent of clinically diagnosed pregnancies end in miscarriage, while three-quarters of them are known as early pregnancy losses, occurring during the first trimester [25,26]. The first trimester of pregnancy comprises the first 14 weeks of pregnancy, correspondingly, the interval from 14 to 28 weeks represents the second trimester [27].

Abortion refers to the termination of a pregnancy, either artificially or naturally, the latter having four recognized forms: threatened, inevitable, total, and missed abortion. Vaginal bleeding in early pregnancy is indicative of a threatened abortion, but a pelvic exam that reveals a closed cervical os, correlated with a transvaginal ultrasound that reveals a live fetus [28]. The inevitable abortion occurs when the cervical os is open during a pelvic exam and there is vaginal bleeding, while on transvaginal ultrasound, a viable fetus may or may not be detected [29]. Abortion is complete when there is initial vaginal bleeding and passage of the fetus through the cervix, correlated with no residual remnants on transvaginal ultrasound [30]. A missed abortion occurs when there is vaginal bleeding and possibly the passage of tissue or pregnancy products, correlated with a closed cervical os, retained products of conception on imaging studies, and no viable fetus [31].

**Figure 1:**
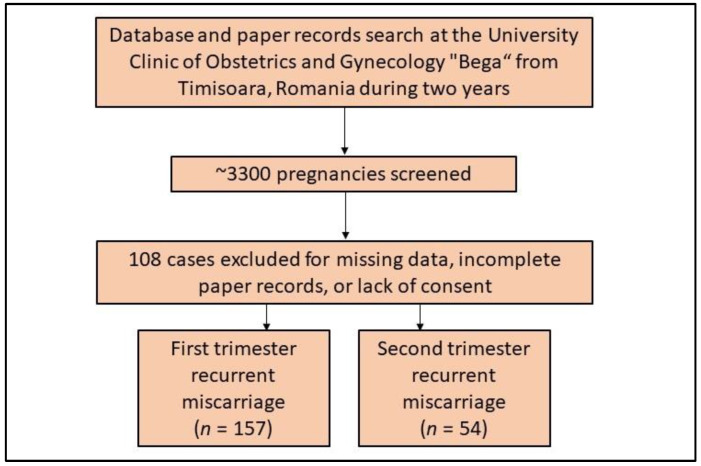
Flowchart displaying cases included in the current study.

The inclusion criteria comprised the following: (1) a history of pregnancy loss; (2) the date of pregnancy loss recorded during the study period; (3) patients giving their consent for their private medical records to be used for research purposes; (4) patients being at least 18 years old; (5) the diagnosis of miscarriage following the previously described definitions; (6) having a thrombophilia test. Patients were excluded from the study according to the following criteria: (1) if medical records were incomplete or there were missing data of interest; (2) when the consent was not signed in the existing papers, as seen in Figure 1; (3) multiple pregnancies were not included. Induced abortion and artificial termination of pregnancy was not considered for analysis as miscarriage. Using a convenience sampling method, it was determined that a total of 287 cases are adequate for findings to be reproducible in the population.

### 2.3. Variables and Data Sources

An electronic database search, and patients’ private records findings contributed to establishing the precise diagnoses of study participants involved, and the status of pregnancy loss in accordance with the International Classification of Diseases (ICD-10). The variables of interest for the current study comprised: (1) maternal background data—age range, body mass index (BMI), area of residence, relationship status, level of income, level of education, occupation, comorbidities, smoking and alcohol use behavior; (2) obstetrical characteristics—gestational age, gravidity, parity, pregnancy-associated complications, history of pregnancy loss, history of abortion (threatened, inevitable, complete, and missed), high obstetrical risk, pelvic infections, history of sexually transmitted diseases (STD), infertility, assisted reproductive techniques; (3) laboratory parameters—factor V Leiden, prothrombin, antithrombin deficiency, protein C deficiency, protein S deficiency, free protein S deficiency, plasminogen activator inhibitor 1 (PAI-1) deficiency, Angiotensin Converting Enzyme (ACE) deletion, Factor VII deficiency, Factor XIII deficiency, β-fibrinogen polymorphism, glycoprotein Ia polymorphism, plasminogen and tissue-type plasminogen activator deficiency, Acquired activated protein C resistance, MTHFR gene mutation. Cases with high obstetrical risk were excluded from the current study to avoid bias risk for pregnancy loss. A high obstetrical risk pregnancy was considered as any condition associated with a pregnancy that creates a significant threat to the mother or fetus [32].

### 2.4. Laboratory Analysis

The samples of whole blood that were taken were placed in vacuum tubes with sodium citrate anticoagulant. Centrifugation for ten minutes yielded platelet-depleted plasma, which was used in the experiment. Immediately after, an analysis was performed on the antithrombin activity. Until the analysis, plasma used to determine other parameters was kept at a temperature of 80 degrees Celsius. The activities of plasma proteins C and S were assessed using a functional clotting assay, while free protein S antigen was determined using an enzyme-linked immunosorbent assay kit. Berichrom was used to determine the level of antithrombin activity, while the lupus-sensitive activated partial thromboplastin time test (APTT), and a dilute Russell’s viper venom time test, were utilized in order to screen for lupus anticoagulant. The latter was carried out in order to provide additional evidence that positive results were obtained. An enzyme-linked immunosorbent test was utilized in order to determine the levels of cardiolipin and 2-glycoprotein antibodies.

In order to make a diagnosis of a deficiency in protein C, protein S, or antithrombin, the percentiles of activity or antigen levels were measured in women. To consistently detect severe deficiencies, we chose the threshold for severe deficiencies to be two-thirds (67%) of the cutoff for the 5th percentile. Using a second blood sample, the levels of antithrombin, protein C, protein S, and free protein S antigen were measured. Standard procedures were followed in order to extract DNA from peripheral blood leukocytes. An allele-specific restriction enzyme test was utilized to determine whether Factor V Leiden (FVL) and prothrombin G20210A mutations were present in the sample under investigation.

### 2.5. Statistical Analysis

Data analysis was performed using the IBM SPSS software version 27.0 (SPSS. Inc., Chicago, IL, USA). Absolute values and their frequencies were used to represent categorical variables. The proportions were analyzed statistically using the Chi^2^ and Fisher’s exact tests. A Shapiro–Wilk test was performed to assess the normality of data and the Student’s t-test was used to compare means of normally distributed variables. A multivariate logistic regression analysis was used to evaluate independent risk variables and the associated odds ratios for pregnancy loss, adjusted by age and body mass index. The significance threshold was set for an alpha value of 0.05.

## 3. Results

### 3.1. Background Characteristics

Table 1 presents the comparison of background characteristics between women with a history of first trimester pregnancy loss and second trimester pregnancy loss. The majority of patients were under 35 years old in both study groups, with only 21.7% overweight and obese in the first trimester group, and 16.7% in the second trimester group, adjusted for gestational age. The substance use behavior identified 4.5% chronic alcohol users and 14.0% smokers among patients with first trimester pregnancy loss, and 5.6% chronic alcohol users and 16.7% smokers in those with second trimester pregnancy loss. The most commonly observed comorbidity in the entire cohort was depression, which was found in approximately 8% of all patients, followed by cardiovascular and metabolic disorders, in approximately 4% of cases. A total of 16 (10.2%) patients had COVID-19 in the first trimester group, compared with 9 (16.7%) patients in the second trimester group, without any significant differences.

### 3.2. Obstetrical Characteristics

The obstetrical characteristics of study participants presented in Table 2 shows that 54.1% of patients in the first trimester had three or more pregnancies, although only 10.2% of all gave birth. In the other study group, a total of 68.5% women had three or more pregnancies, and only 9.3% had a child. The difference in proportions was not statistically significant. The studied patients suffered a total of 496 spontaneous abortions, with a statistically significant difference in proportions when comparing the types. Therefore, 18.5% missed abortions happened in the first trimester, compared with only 6.2% in the second trimester (*p*-value = 0.003). The most common type was a complete abortion that occurred in 39.1% of first trimester pregnancy losses, and 49.1% in the second trimester. The history of induced abortions was not statistically significant between study groups. However, high obstetrical risk was a major finding in patients who suffered second trimester pregnancy losses (38.9% vs. 21.0% in the first trimester, *p*-value = 0.009). Among pregnancy-related complications, only the proportion of maternal infections was statistically significantly different between study groups (27.8% in the second trimester, compared with 15.3% in the first trimester, *p*-value = 0.041).

### 3.3. Laboratory Analysis

The analysis of thrombophilia factors was mostly insignificant when comparing first and second trimester pregnancy losses, although four factors were identified as being more prevalent in one of the groups. Therefore, the Factor V Leiden homozygosity was statistically significantly more common in pregnant women who suffered first trimester pregnancy losses, compared with those who had second trimester miscarriages (10.8% vs. 1.9%, *p*-value = 0.041), as seen in Table 3. In the first trimester group, the presence of antiphospholipid syndrome antibodies was also a significantly more common finding compared with second trimester pregnancy losses (17.8% vs. 5.6%, *p*-value = 0.027). On the other hand, Protein C deficiency and Glycoprotein Ia polymorphism were statistically significantly more frequent in the second trimester group (14.8% vs. 5.7%, *p*-value = 0.034; 33.3% vs. 19.7%, *p*-value = 0.041).

### 3.4. Risk Factor Analysis

**Figure 2:**
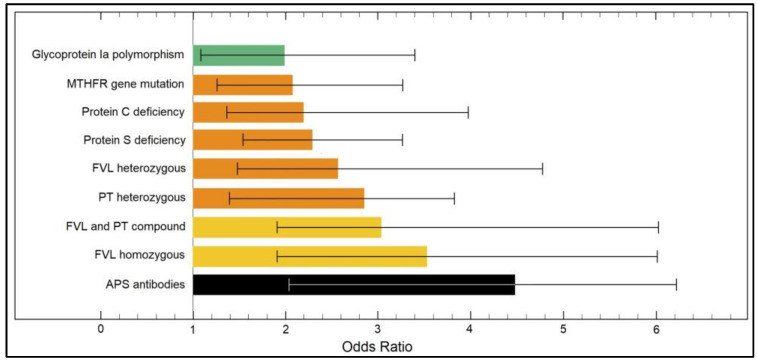
Risk factor analysis for early (first trimester) pregnancy loss.

**Figure 3:**
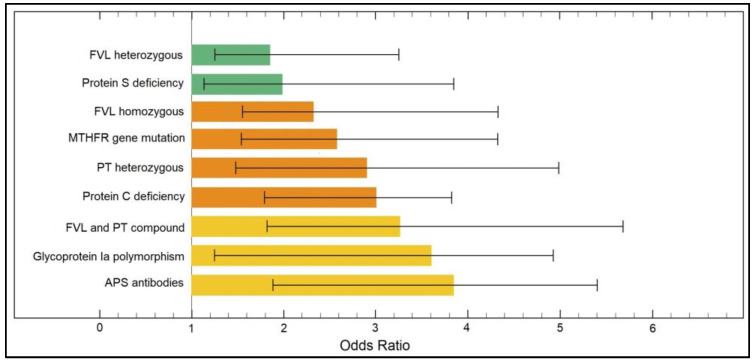
Risk factor analysis for late (second trimester) pregnancy loss.

The multivariate risk factor analysis presented in Table 4 determined a series of significant risk factors from the panel of thrombophilia mutations and deficiencies for both first trimester and second trimester pregnancy losses. The strongest independent risk factors for first trimester pregnancy loss were FVL and PT compound mutations (OR = 3.11,), followed by FVL homozygous mutation (OR = 3.66), and APS antibodies (OR = 4.47), as described in Figure 2. Regarding second trimester pregnancy loss risk factors, the strongest were FVL and PT compound (OR = 3.24), followed by Glycoprotein Ia polymorphism (OR = 3.61) and APS antibodies (OR = 3.85), as presented in Figure 3.

## 4. Discussion

### 4.1. Important Findings

The current study identified valuable information about the involvement of thrombophilia in recurrent pregnancy loss. The novelty of the study stands in identifying separately particular thrombophilia factors responsible for first and second trimester pregnancy loss. It was observed that FVL and PT compound mutations, followed by FVL homozygous mutation, and APS antibodies were the predominant risk factors for first trimester pregnancy loss. Similar findings as FVL and PT compound mutations, followed by Glycoprotein Ia polymorphism and APS antibodies were the main risk factors for second trimester pregnancy loss. Additionally, this study now addresses a large number of issues that can help guide management decisions regarding the necessity of thromboprophylaxis. It does so by quantifying the number of heritable thrombophilia factors in correlation with episodes of thromboembolism during pregnancy and the number of pregnancies lost.

Existing guidelines for prenatal thromboprophylaxis in women with heritable thrombophilia is noticeably inconsistent due to a lack of knowledge regarding the interaction with a family history of thromboembolism, and varying perceptions of the risk threshold above which pharmacologic prophylaxis is deemed appropriate [33]. In homozygous carriers of FVL, there are scarce data available regarding the risk of pregnancy-associated venous thromboembolism (VTE). One study identified a probability of 3.4% for VTE associated with homozygosity for FVL in pregnant women [18]. One systematic review found a similar risk of VTE in pregnant women with inherited thrombophilia, but not necessarily with a family history of VTE, and reported probability of 4.8% [34].

According to one study, a compound defect consisting of heterozygous FVL and prothrombin G20210A is linked to a disproportionately increased risk in comparison to the risk posed by each mutation taken separately [35]. It was also found that a positive family history of venous thromboembolism (VTE) raises the risk of venous thromboembolism by a factor of two to four, independent of the presence or type of thrombophilia [7,36]. However, a positive family history of venous thromboembolism in first-degree relatives was not a factor in the relative risk of thrombosis that was related with homozygous deficiencies or compound heterozygous FVL and prothrombin G20210A.

It is debatable whether mutations in antithrombin or proteins C and S are relevant, and even more precisely, whether they have any prognostic value. As was to be expected, minor deficiencies, which constitute the majority of clinical cases, such as antithrombin activity lower 90% or protein C activity under 76%, are linked with a less obvious increase in risk in comparison to severe deficiencies [18,37].

In the context of early pregnancy miscarriage, one review discovered that the prevalence of inherited thrombophilia in a large cohort of women with a history of early three or more miscarriages is comparable to that of the general population, and that the prevalence of acquired thrombophilia is low and does not significantly differ from the prevalence reported in the general population. Both of these findings pertain to the fact that the incidence of inherited thrombophilia is similar to that of the general population. Empirical testing and treatment for thrombophilia, such as low dose aspirin and low molecular heparin, should not be advised, unless there is unequivocal clinical and laboratory evidence of established disease. This is because the contribution of inherited and acquired thrombophilia in causing early recurrent miscarriage is extremely low; therefore, pregnant women and their clinical practitioners should be aware of this fact [38,39].

Although many thrombophilia mutations were identified among the patients included in this study, the exact etiology of miscarriage is still unknown due to the lack of data such as the presence of aneuploidy. It is not generally suggested to perform parental karyotyping, only after conducting an individual risk assessment should the possibility be considered, given the extremely low likelihood of discovering an abnormality [40]. In European populations, the average age of a woman when giving birth for the first time is close to 30 years old, while it is known that advanced female age is associated with an increased chance of embryonic aneuploidy. As a result, embryonic aneuploidy will often be the cause of RPL, particularly in women older than 36 years old [41]. The age of the female patient and the number of previous pregnancies that were unsuccessful, in addition to other maternal conditions such as a manifest autoimmune or coagulative disease, family history, and the results of any miscarriage tissue karyotyping that may have been performed, should all be considered when deciding when to begin investigations [42]. In addition to that, it should be the outcome of collaborative decision making between the couple and the doctor, all while being in accordance with the resources that are now accessible. It is recommended that individualized diagnostic testing be explored, in which some tests may be performed, while others are skipped.

### 4.2. Study Limitations

Among study limitations, it should be noted that a larger sample size may be necessary to generate sufficient statistical power; however, analyzing the first and second trimester RPL was sufficient using this small sample size since the findings found were consistent with past research in this field. In fact, these results shed more light on the significance of testing for FVL mutation in women with a history of pregnancy loss in order to determine the function of anticoagulants in recurrent pregnancy loss. Another limitation of the current study is the lack of a control group with thrombophilia and successful pregnancies. The reason behind this is the high cost of laboratory and genetic analysis for thrombophilia that is not covered by the government or insurance. Additionally, there were no data on the etiology of miscarriages such as aneuploidies or infections.

## 5. Conclusions

Various thrombophilia risk factors were identified for early and late pregnancy loss, including several mutations that seem to affect fetal development, particularly during the first or second trimester. However, it is currently not suggested that women who have not previously had difficulties during pregnancy undergo regular testing for thrombophilia abnormalities. Despite this, the avoidance of miscarriage, early and late-onset fetal growth restriction, and stillbirth continues to be a significant and present concern in the field of public health. Concerning hereditary thrombophilia associated with early or late pregnancy loss as well as other pregnancy-related issues, it is presently unclear if the process itself as well as the natural history of the condition is fully known. Due to the rarity of hereditary thrombophilia in the general population, previous research on the subject was often underpowered to identify any meaningful findings, including the results of the current investigation. Even though thrombophilia screening is risk-free and efficient, there is no intervention that has been shown to be effective after screening to decrease the rate of recurrent pregnancy loss.

## Figures and Tables

**Table 1:** Comparison of patients’ socio-demographic and clinical characteristics.

Variables	First Trimester (*n* = 157)	Second Trimester (*n* = 54)	*p*-Value *
Age (≥ 35 years)	71 (45.2%)	22 (40.7%)	0.567
BMI (>25 kg/m^2^) **	34 (21.7%)	9 (16.7%)	0.432
Area of residence (urban)	112 (71.3%)	33 (61.1%)	0.162
Relationship status (married)	136 (86.6%)	46 (85.2%)	0.791
Level of income (average or higher)	98 (62.4%)	33 (61.1%)	0.864
Level of education (higher education)	92 (58.6%)	36 (66.7%)	0.295
Occupation (employed)	157 (80.3%)	42 (77.8%)	0.696
**Substance use behavior**			
Frequent alcohol consumption	7 (4.5%)	3 (5.6%)	0.743
Frequent smoker	22 (14.0%)	9 (16.7%)	0.634
**Chronic comorbidities**			
Cardiovascular	5 (3.2%)	2 (3.7%)	0.854
Metabolic	6 (3.8%)	4 (7.4%)	0.284
Autoimmune	1 (0.6%)	2 (3.7%)	0.100
Respiratory	5 (3.2%)	1 (1.9%)	0.611
Other	2 (1.3%)	1 (1.9%)	0.756
History of depression	11 (7.0%)	5 (9.3%)	0.589
History of COVID-19	16 (10.2%)	9 (16.7%)	0.204

* Chi-square or Fisher’s exact test; ** Weight measured when pregnancy loss occurred; BMI—Body mass index.

**Table 2:** Comparison of obstetrical characteristics between women with a history of first trimester and second trimester pregnancy loss.

Variables	First Trimester (*n* = 157)	Second Trimester (*n* = 54)	*p*-Value *
**Gravidity**			0.179
1	28 (17.8%)	7 (13.0%)	
2	44 (28.0%)	10 (18.5%)	
≥3	85 (54.1%)	37 (68.5%)	
Parity (1 or more)	16 (10.2%)	5 (9.3%)	0.843
**History of pregnancy loss**			0.336
1	8 (5.1%)	4 (7.4%)	
2	41 (26.1%)	9 (16.7%)	
≥3	108 (68.8%)	41 (75.9%)	
**Spontaneous abortion (*n* = 496)**			0.003
Threatened	34 (10.1%)	17 (10.6%)	
Inevitable	107 (31.7%)	55 (34.2%)	
Complete	132 (39.1%)	79 (49.1%)	
Missed	62 (18.5%)	10 (6.2%)	
**History of induced abortion (*n* = 37)**			0.077
Medical	14 (66.7%)	6 (37.5%)	
Surgical	7 (33.3%)	10 (62.5%)	
High obstetrical risk	33 (21.0%)	21 (38.9%)	0.009
**Pregnancy-related complications**			
Preeclampsia	9 (5.7%)	3 (5.6%)	0.961
Anemia	23 (14.6%)	9 (16.7%)	0.721
Peripartum infection	12 (7.6%)	6 (11.1%)	0.431
Other maternal infections	24 (15.3%)	15 (27.8%)	0.041
Deep venous thrombosis	23 (14.6%)	12 (22.2%)	0.196
Pulmonary embolism	5 (3.2%)	2 (3.7%)	0.854
Other episodes of thrombosis	17 (10.8%)	8 (14.8%)	0.434
**Female infertility**			
Assisted reproductive techniques	39 (24.8%)	11 (20.4%)	0.505
History of STDs	18 (11.5%)	9 (16.7%)	0.323
Pelvic infections	25 (15.9%)	12 (22.2%)	0.293

* Chi-square or Fisher’s exact test; STD—Sexually transmitted disease.

**Table 3:** Comparison of laboratory analysis between women with a history of first trimester and second trimester pregnancy loss.

Variables	First Trimester (*n* = 157)	Second Trimester (*n* = 54)	*p*-Value *
FVL heterozygous	36 (22.9%)	10 (18.5%)	0.498
FVL homozygous	17 (10.8%)	1 (1.9%)	0.041
PT heterozygous	11 (7.0%)	6 (11.1%)	0.339
FVL and PT (compound heterozygous)	15 (9.6%)	4 (7.4%)	0.634
Antithrombin deficiency	13 (8.3%)	4 (7.4%)	0.838
Protein C deficiency	9 (5.7%)	8 (14.8%)	0.034
Protein S deficiency	14 (8.9%)	6 (11.1%)	0.634
Free protein S deficiency	7 (4.5%)	5 (9.3%)	0.188
PAI-1 deficiency	2 (1.3%)	1 (1.9%)	0.756
ACE deletion	9 (5.7%)	3 (5.6%)	0.961
Factor VII deficiency	16 (10.2%)	4 (7.4%)	0.546
Factor XIII deficiency	13 (8.3%)	7 (13.0%)	0.310
β-fibrinogen polymorphism	29 (18.5%)	9 (16.7%)	0.765
Glycoprotein Ia polymorphism	31 (19.7%)	18 (33.3%)	0.041
tPA deficiency	10 (6.4%)	4 (7.4%)	0.791
APCR	4 (2.5%)	1 (1.9%)	0.771
APS antibodies **	28 (17.8%)	3 (5.6%)	0.027
MTHFR gene mutation	32 (20.4%)	12 (22.2%)	0.774

* Chi-square or Fisher’s exact test; ** Anticardiolipin antibodies IgG or IgM (ELISA), Anti-beta-2-glycoprotein-I antibodies IgG or IgM (ELISA), Lupus anticoagulants; PT—prothrombin; FVL—Factor V Leiden; MTHFR—Methylene tetrahydrofolate reductase; ACE—Angiotensin Converting Enzyme Deletion; APCR—Acquired activated protein C resistance; PAI-1—plasminogen activator inhibitor 1; tPA—plasminogen and tissue-type plasminogen activator deficiency; APS—antiphospholipid syndrome.

**Table 4:** Risk factor analysis for first and second trimester pregnancy loss.

Risk Factors *	First Trimester Pregnancy Loss (OR–95% CI)	*p*-Value	Second Trimester Pregnancy Loss (OR–95% CI)	*p*-Value
FVL heterozygous	2.54 (1.33–4.96)	0.036	1.82 (1.24–3.25)	0.049
FVL homozygous	3.66 (1.85–6.11)	0.001	2.27 (1.51–3.88)	0.007
PT heterozygous	2.79 (1.27–3.82)	0.022	2.81 (1.58–4.33)	0.001
FVL and PT compound	3.11 (1.89–6.18)	0.001	3.24 (1.80–5.76)	0.001
Protein C deficiency	2.15 (1.32–3.93)	0.009	2.98 (1.75–5.04)	0.001
Protein S deficiency	2.25 (1.46–3.23)	0.001	1.93 (1.16–2.83)	0.012
APS antibodies	4.47 (2.03–6.32)	0.001	3.85 (1.83–5.41)	0.001
MTHFR gene mutation	2.02 (1.24–3.32)	0.017	2.48 (1.37–4.29)	0.001
Glycoprotein Ia polymorphism	1.97 (1.08–3.40)	0.033	3.61 (1.22–4.94)	0.001

* Data adjusted for age and body mass index; CI—Confidence Interval; OR—Odds Ratio.

## Data Availability

The data presented in this study are available on request from the corresponding author.
